# Expression of MEP Pathway Genes and Non-volatile Sequestration Are Associated with Circadian Rhythm of Dominant Terpenoids Emission in *Osmanthus fragrans* Lour. Flowers

**DOI:** 10.3389/fpls.2017.01869

**Published:** 2017-10-30

**Authors:** Riru Zheng, Cai Liu, Yanli Wang, Jing Luo, Xiangling Zeng, Haiqin Ding, Wei Xiao, Jianping Gan, Caiyun Wang

**Affiliations:** ^1^Key Laboratory for Biology of Horticultural Plants, Ministry of Education, College of Horticulture and Forestry Sciences, Huazhong Agricultural University, Wuhan, China; ^2^Key Laboratory of Urban Agriculture in Central China, Ministry of Agriculture, Wuhan, China; ^3^Hubei Key Laboratory of Economic Forest Germplasm Improvement and Resources Comprehensive Utilization, Hubei Collaborative Innovation Center for the Characteristic Resources Exploitation of Dabie Mountains, Huanggang Normal University, Huanggang, China

**Keywords:** *Osmanthus fragrans* Lour., terpenoid, circadian emission, MEP pathway, glycosylation

## Abstract

*Osmanthus fragrans* Lour. is one of the top 10 traditional ornamental flowers in China famous for its unique fragrance. Preliminary study proved that the terpenoids including ionone, linalool, and ocimene and their derivatives are the dominant aroma-active compounds that contribute greatly to the scent bouquet. Pollination observation implies the emission of aromatic terpenoids may follow a circadian rhythm. In this study, we investigated the variation of volatile terpenoids and its potential regulators. The results showed that both volatile and non-volatile terpenoids presented circadian oscillation with high emission or accumulation during the day and low emission or accumulation during the night. The volatile terpenoids always increased to reach their maximum values at 12:00 h, while free and glycosylated compounds continued increasing throughout the day. The depletion of non-volatile pool might provide the substrates for volatile emission at 0:00–6:00, suggesting the sequestration of non-volatile compounds acted like a buffer regulating emission of terpenoids. Further detection of MEP pathway genes demonstrated that their expressions increased significantly in parallel with the evident increase of both volatile and non-volatile terpenoids during the day, indicating that the gene expressions were also closely associated with terpenoid formation. Thus, the expression of MEP pathway genes and internal sequestration both played crucial roles in modulating circadian rhythm of terpenoid emission in *O. fragrans*.

## Introduction

Most plants release diverse blends of low molecular weight and high vapor pressure organic compounds from flowers, leaves and fruits into the atmosphere. These volatile compounds represent approximately 1% of the whole secondary metabolites (Dudareva et al., 2004). By emitting aromatic compounds from flowers, plants attract pollinators and repel antagonists to serve plant fitness (Yu et al., 2014; Florian, 2015). In addition, these aromatic compounds significantly promote ornamental value and are utilized by modern fragrance industry (Wang et al., 2009; Yue et al., 2014).

*Osmanthus fragrans* Lour. is one of the top 10 traditional plants in China. It has a long cultivation history and famous for its unique fragrance (Xiang and Liu, 2008). Its aromatic flowers are important primary material of expensive perfumes, flavorings, and cosmetics (Wang et al., 2009). To date, extensive attention has been paid to analyze its volatile aroma compounds. More than 70 volatile compounds have been detected in fresh flowers and essential oil and they are mainly composed of terpenoids, C6 compounds, and esters (Wang et al., 2009; Baldermann et al., 2010; Xin et al., 2013). The terpenoids including linalool and its derivatives, β-ocimene and β-ionone widely exist in most varieties and are identified as characteristic active-aromatic compounds in fresh flowers by GC-O technology (Cai et al., 2014). It is also proved that linalool and its oxides, α-ionone, and β-ionone are important extracts in essential oil of *O. fragrans* (Wang et al., 2009). Emission of terpenoids in many species is highly regulated by internal metabolic processes and displays a diurnal/nocturnal rhythm (Aharoni et al., 2003; Simkin et al., 2004). Pollination observation of *O. fragrans* revealed that insects including *Vespa nigrithorax, Apis florea*, and *Episyrphus balteatus* were the dominant pollinators and only visited flowers during the day with peak visiting frequency from 9:00 to 12:00 h (Zhang, 2013). Since the floral scent bouquet is one of the most important cues for attracting pollinators (Yang et al., 2013; Florian, 2015), this phenomenon strongly implies that the emission of aromatic terpenoids may follow a coordinated rhythmic oscillation in *O. fragrans*. However, the specific circadian rhythm of volatile terpenoids emission and corresponding regulator along with potential biological function remain unclear.

To date, numerous studies deciphered that the emission or/and formation of a wide range of aroma compounds was confined to diurnal/nocturnal cycle due to the control of relevant genes expression (Dudareva et al., 2005). For example, in *Antirrhinum majus* flowers, a free-running internal circadian clock regulated the monoterpene and methylbenzoate synthase gene expression and corresponding emission of volatile aroma compounds (Dudareva et al., 2003). Terpenoids such as β-ionone presented rhythmic emission coinciding with the carotenoid cleavage dioxygenase gene (*PhCCD1*) expression in *Petunia hybrida* flowers (Simkin et al., 2004). In *O. fragrans*, linalool and ocimene are synthesized through MEP pathway which is cooperated by a chain of genes including *DXS, DXR, CMK, MCT, MECPS, HDS, IDS, IDI, GPPS*, and various *TPSs* (Dudareva et al., 2013). α-Ionone and β-ionone are cleavage products by OfCCD1 and OfCCD4 enzymes using carotenoid as substrate which originates from geranylgeranyl pyrophosphate (GGPP) (Baldermann et al., 2010). Whether the genes involved in MEP pathway are responsible for the circadian emission of important terpenoids is not clear. However, lack of transcriptome information has severely inhibited the explore of relationship between them. Thus, in this paper, a transcriptome sequencing was first carried out based on Illumina HiSeq 2000 platform and a series of MEP pathway genes were selected.

It is also worth noticing that relevant gene expression is not the only important factor of aroma compound release. Although most previous studies in *O. fragrans* predominantly focused on the volatile compounds, a considerable portion of aroma compound can be stored as free compounds or/and be transformed to glycosylated compounds and some evidence suggests the internal sequestration can exert significant influence on the emission of volatile compounds (Bönisch et al., 2014; Yar-Khing et al., 2014). In *Actinidia chinensis* flowers, its primary volatile terpenes *(S)-(E)*-nerolidol, (*E, E*)-farnesol and *(S)*-linalool could all be accumulated as glycoside and a majority of 8-hydroxylinalool glycoside even predominantly accumulated in tissue leading to a remarkable decline in the *(S)*-linalool emission (Green et al., 2012). In *O. fragrans*, glycoside compounds were detected in fresh flowers by adding β-D-glucosidase, however, the effect of non-volatile sequestration on terpenoids emission is still rudimentary (Yang et al., 2005; Zeng et al., 2016).

In this study, the circadian emission of important terpenoids in *O. fragrans* was investigated in detail. In order to better understand the potential association between emission and sequestration, free and glycosylated terpenoids were detected as well. Furthermore, the expressions of relevant MEP pathway genes were examined to elucidate whether they were relative with the circadian emission of terpenoids. The analysis on aroma-active terpenoids in *O. fragrans* may not only help us to understand its potential biological function but also provide a solid foundation for further genetic modification of floral scent.

## Materials and Methods

### Plant Material

*Osmanthus fragrans* ‘Liuye Jingui’ was cultivated in the nursery of Huazhong Agricultural University in Wuhan, Hubei Province, China. The whole process of flower opening is divided into four stages (Zou et al., 2014): tight bud stage, initial blossoming stage, full blossoming stage, and late blossoming stage. Flowers at four stages were collected and equivalently mixed as samples for transcriptome sequencing. To minimize the effect of developmental regulation on aroma compounds, the relatively stable full blossoming period (3- to 5-day-old) was selected for analysis of circadian rhythm. The flower samples were collected at 6:00, 12:00, 18:00, and 0:00 h for successive 3 days, respectively. Part of the fresh samples were directly used for volatile compound analysis, and the remainder were immediately frozen in liquid nitrogen and stored -80°C for solvent, glycoside extraction, transcriptome sequencing, and gene expression detection. Each experiment contained three biological repeats.

### Headspace Volatiles Collection, Solvent Extraction, and Glycoside Extraction

Headspace volatiles were collected according to previous studies (Cai et al., 2014; Zeng et al., 2016). Two-gram of fresh flowers were sealed in a 20 ml capped solid-phase microextraction (SPME) vial and incubated at 25 ± 2°C for 30 min. SPME fiber (50/30 μm DVB/CAR/PDMS on a 2 cm StableFlex fiber, Supleco Inc., Bellefonte, PA, United States) was then exposed to the headspace of the capped vial for 30 min. The fiber was manually injected at the port of the gas chromatograph (GC) for desorption at 230°C for 5 min in splitless mode.

Solvent extractions were carried out according to previous methods (Green et al., 2012). Two-gram of frozen flowers were ground to a fine powder in liquid N_2_ and then transferred to a 50 ml centrifuge tube. The powder was extracted twice in 10 ml pentane/Et_2_O (1:1 v/v) mixture for 30 min at 16°C. The two extractions were combined and stored overnight at -20°C. The following day, the upper solvent layer was carefully separated and reduced to 2 ml by a gentle stream of N_2_. The concentrated extract, with 47.3 mg/μl cyclohexanone added as internal standard, was passed through a column of anhydrous MgSO_4_ to remove any remaining water and then injected into the GC for further detection.

Glycoside analysis was also carried out according to previous methods (Green et al., 2012). Two-gram of frozen flowers were ground to a fine powder in liquid N_2_ and resuspended in 30 ml ddH_2_O. The sample was centrifuged at 8,000 × *g* for 15 min at 4°C twice. The supernatant run on a 15 mm × 25 mm i.d. Amberlite XAD-2 column (Supelco, Bellefonte, PA, United States) to remove the soluble sugars and acids with water and pentane/Et_2_O (1:1 v/v), respectively. The bound glycosides were eluted with 20 ml methanol and evaporated to dryness in a rotary evaporator. The glycoside pellet was resuspended in 2 ml of de-glycosylation buffer (200 mM Na_2_HPO_4_, 220 mM citric acid, pH 5.0) and re-extracted three times with 1 ml pentane/Et_2_O (1:1 v/v) to remove non-glycosylated compounds. The 300 μl β-D-glucosidase (≥6 μ/mg) (Sigma-Aldrich Co., LLC, United States) and 10 μl cyclohexanone (concentration of 9.46 mg/ml soluble in methanol) were added for enzymatic hydrolysis and as internal standard, respectively, covered by 1 ml MTBE. The hydrolysis samples were overlaid with 1 ml pentane/Et_2_O (1:1 v/v) and incubated at 40°C for 36 h. After that, the samples were extracted with 1 ml pentane/Et_2_O (1:1 v/v) three more times. All the extracts passed through a column of anhydrous MgSO_4_ and reduced to 2 ml under a gentle stream of N_2_.

### GC-MS Analysis

The collected headspace, extracted solvent, and glycoside samples were then subjected to GC-MS test according to our previous study (Cai et al., 2014; Zeng et al., 2016). The identification of the aroma compounds was based on a comparison of their mass spectra, retention indices (RIs) with the authentic standards (C8-C40 alkane standard solution, linalool and β-ocimene) and mass spectra database in the National Institute of Standards and Technology (NIST, version 2.0d, 2005). Peaks were selected and integrated by the molecular ion and/or specific diagnostic ions of each compound. The calibration curves of target analysis combined with the internal standard method were used to quantify these compounds.

### Construction of Transcriptome Library and Gene Function Annotation

The 0.2 g frozen flowers from the four-stage samples were used for RNA extraction and an equally mixed of 3 μg RNA was applied as input material for the RNA sample preparations. Sequencing libraries were generated using NEBNext^®^ Ultra^TM^ RNA Library Prep Kit for Illumina^®^ (NEB, United States) following manufacturer’s recommendations and index codes were added to attribute sequences to each sample. The library preparations were sequenced on an Illumina HiSeq 2000 platform and paired-end reads were generated. Clean data (clean reads) were obtained by removing reads containing adapter, reads containing ploy-N and low quality reads from raw data. Moreover, Q20, Q30, GC-content and sequence duplication level of the clean data were calculated. All the downstream analyses were based on clean data with high quality. Transcriptome assembly was accomplished based on the left.fq and right.fq using Trinity (Grabherr et al., 2011) with min_kmer_cov set to 2 by default and all other parameters set default.

Gene function was annotated based on the following databases: Nr (NCBI non-redundant protein sequences), Nt (NCBI non-redundant nucleotide sequences), Pfam (Protein family), KOG/COG (Clusters of Orthologous Groups of proteins), Swiss-Prot (A manually annotated and reviewed protein sequence database), KO (KEGG Ortholog database), and GO (Gene Ontology).

### qRT-PCR for Gene Expression Analysis

The samples collected at 6:00, 12:00, 18:00, and 0:00 h during the full blossoming period were subjected to qRT-PCR tests to determine the transcript abundance of the genes involved in formation of terpenoids. The experiments were performed on Applied Biosystems 7500 Fast Real-Time PCR platform using the SYBR^®^ Premix Ex Taq^TM^ II mix (Takara Biotechnology Co., Ltd., Dalian, Japan) and the results were analyzed by the Applied Biosystems 7500 software (Applied Biosystems Life Technologies). Three biological replicates were tested and relative transcript levels were calculated by the 2^-ΔΔC_T_^ method using *β-Actin* as the endogenous control gene for data normalization. The relative gene expression was determined as previously described by Livak and Schmittgen (2001). The primers for qRT-PCR analysis are listed in Supplementary Table S1.

## Results

### Circadian Rhythm of Three Major Terpenoids in *O. fragrans*

**FIGURE 1:**
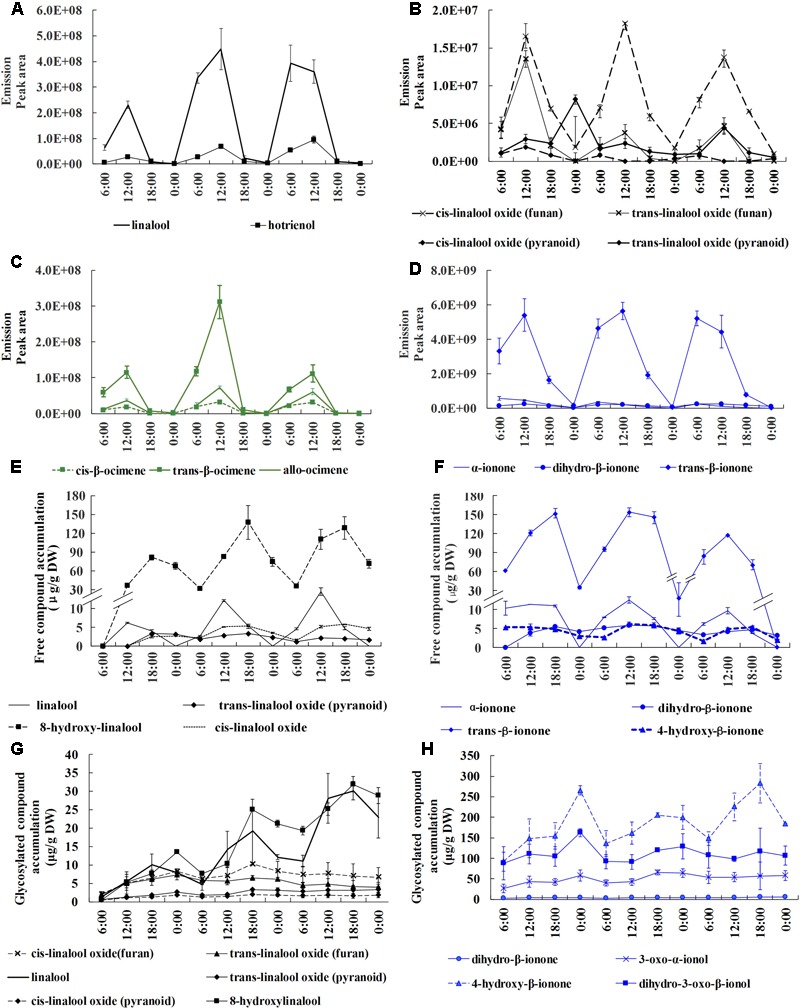
Circadian variation of volatile, free and glycosylated terpenoids during the full blossoming period in *O. fragrans* flowers. **A–D** represent emission of volatile terpenoids, **E** and **F** represent accumulation of free terpenoids, **G** and **H** represent accumulation of glycosylated terpenoids. **(A,B)** Emission of linalool and its derivatives. **(C)** Emission of ocimene and its derivatives. **(D)** Emission of ionone and its derivatives. **(E)** Accumulation of free linalool and its derivatives. **(F)** Accumulation of free ionone and its derivatives. **(G)** Accumulation of glycosylated linalool and its derivatives. **(H)** Accumulation of glycosylated ionone and its derivatives. Results represent the mean ± SE of three technical repetitions and three biological replicates.

To investigate the circadian rhythmicity of these aroma compounds, we conducted volatile, free and glycosylated aroma compound analyses at 6 h intervals throughout the relatively stable full blossoming period. Monoterpenes (linalool, ocimene) and their derivatives along with carotenoid-derived components (ionone and its derivatives) were detected as the principal terpenoids in *O. fragrans*. A diversity of derivatives produced from ionone, linalool, ocimene widely exist in volatile, free and glycosylated forms. Linalool produced 6 derivatives, including *cis*-linalool oxide (furan), *trans*-linalool oxide (furan), *cis*-linalool oxide (pyran), *trans*-linalool oxide (pyran), hotrienol and 8-hydroxylinalool. Linalool and its oxides widely existed in volatile, free and glycosylated forms, however, hotrienol only detected in volatile form and 8-hydroxylinalool detected in free and glycosylated forms. Ionone also had rich varieties, however, only dihydro-β-ionone could be found in three forms, α-ionone and trans-β-ionone could be detected in volatile and free forms and 4-hydroxyl-β-ionone in free and glycosylated forms. Ocimene mainly included volatile *trans*-β-ocimene, cis-β-ocimene and allo-ocimene without any free and glycosylated forms (**Figure 1**). A proportion of compounds with high volatility were easily released and others were sequestered in non-volatile free and/or glycosylated forms in flowers.

Since the terpenoids were proved to be the key characteristic aroma compounds in sweet osmanthus, detailed analyses were conducted to elucidate the circadian discipline of terpenoids. All volatile terpenoids presented a drastic oscillation with abundant emission during day and scarce emission during night. Maximum emission uniformly and repeatedly appeared at 12:00 h in all terpenoids throughout the whole full-blossoming period. Total of three volatile terpenoids rose up to the maximum value at 7.03^∗^10^9^ and reduced to the minimum value at 1.04^∗^10^9^. In terms of volatile amount, trans-β-ionone, linalool and *trans*-β-ocimene were the main volatile compounds. Trans-β-ionone was the most dominant volatile compounds with its maximum peak area at more than 5^∗^10^9^, approximately 10 times higher than the following linalool and trans-β-ocimene (**Figures 1 A–D**). Although an amazing diversity of linalool derivatives could be found in volatile profiles, they only accounted for a low proportion compared to linalool itself (**Figures 1A,B**).

Free compounds showed a coordinated and lagging rhythm when compared with volatile compounds. Total free compounds also increased during the day and decreased during the night. The accumulation of majority free compounds constantly increased till 18:00 h, 6 h later than the volatile form, suggesting the terpenoids persistently formed during 12:00–18:00 h. Despite the simultaneous increase during the day, an opposite variation could be observed between volatile and free compounds at night. A rapid increase of volatile terpenoids occurred at 0:00–6:00 h, in contrast to a considerable decrease of free terpenoids, indicating that the depletion of free compounds provided the substrate for emission. High volatile compounds such as linalool which took lead in the volatile form was barely detected in free form, whereas high molecular weight compound such as 8-hydroxylinalool was significantly accumulated. *Trans*-β-ionone was still the most principal in free form, reaching its peak value at 154.11 μg/g DW, followed by 8-hydroxylinalool with peak value at 138.03 μg/g DW (**Figures 1E,F**).

Glycosylated compound profiles also displayed circadian rhythm in parallel with volatile and free compounds. Constant increases were observed from 6:00 to 18:00 and unified decreases appeared from 0:00 to 6:00 repeatedly, suggesting the accumulation of glycosides was intrigued by formation of aroma compounds during the day. The glycosylated compounds were different from the volatile and free compounds to some extents. For example, *trans*-β-ionone that accounted for the most proportion in both volatile and free forms was not detected in the glycosylated form. Instead, 4-hydroxyl-β-ionone, dihydro-3-oxo-β-ionol and 3-oxo-α-ionol were the predominant compounds, suggesting that ionone was processed to be different derivatives before glycosylation (**Figure 1H**). The content of glycosylated ionone derivatives could reach the maximum content at 283.39 μg/g DW, significantly higher than linalool and its derivatives at 31.94 μg/g DW (**Figures 1G,H**). We also detected a large number of glycoside esters, benzenoid and their derivatives, however, since they were non-volatile and hardly contributed to the odor sense, detailed analysis was not conducted.

Based on the comprehensive data of volatile and non-volatile terpenoids, it was obvious that the formation of terpenoids were activated during the day and simultaneous increases were observed in volatile, free, and glycosylated terpenoids from 6:00 to 12:00. Since then the volatile terpenoids decreases significantly, while the free and glycosylated continued to increase till 18:00 or 0:00. The formation of terpenoids declined during the night and a considerable proportion of free or/and glycosylated terpenoids were transformed into volatile terpenoids at 0:00–6:00. Thus, the circadian emission of volatile terpenoids was closely associated with the variation of non-volatile terpenoids.

### Transcriptome Sequences Analysis and Identification of Relevant Genes Involved in Terpenoid Metabolism

Transcriptome sequencing of flowers in ‘Liuyejingui’ yielded 49,995,711 raw reads and 47,761,953 clean reads (4.78G) (Supplementary Table S2). The clean reads assembled into 85,258 transcripts and 49,781 unigenes, with an average length of 1005 and 760 bp, respectively (Supplementary Table S3). The Q20 reached more than 97% and the GC ratio was approximately 43% (Supplementary Table S2). All the assembled unigenes were aligned by blastx to the databases and a total of 29,311 unigenes were annotated (Supplementary Table S4).

The 20,849 unigenes were assigned with GO terms and 1,646 unigenes were identified and divided into three main categories (**Supplementary Figure S1**). According to KOG, 11,273 assembled unigenes were mainly distributed in general function (1677 unigenes, 14.88%), posttranslational modification (1372 unigenes, 12.17%), protein turnover and chaperones (968 unigenes, 8.59%) among 26 categories (**Supplementary Figure S2**). The 9,926 unigenes were assigned to five main categories base on KEGG, including cellular processes (1,029 unigenes, 10.37%), environmental information processing (866 unigenes, 8.72%), genetic information processing (2,070 unigenes, 20.85%), metabolism (4,329 unigenes, 43.61%), organismal system (1,632 unigenes, 16.44%). It revealed that 232 unigenes were involved in metabolism of terpenoids and polyketides (**Supplementary Figure S3**). In addition, based on transcriptome sequencing, we isolated 19 important relevant genes, including 14 upstream genes in MEP pathway, 3 TPSs genes (*OfLIS1, OfLIS2, OfOCI*), and 2 carotenoid cleavage dioxygenases genes (*OfCCD1* and *OfCCD4*). OfLIS1, OfLIS2, and OfOCI are reported to produce β-linalool and *trans*-β-ocimene through MEP pathway (Zeng et al., 2016), and OfCCD1 and OfCCD4 cleave carotenoid to yield α-ionone and β-ionone (Baldermann et al., 2010) in *O. fragrans*. The sequencing lays the foundation for further analysis.

### Detection of Relevant Genes Expression

**FIGURE 2:**
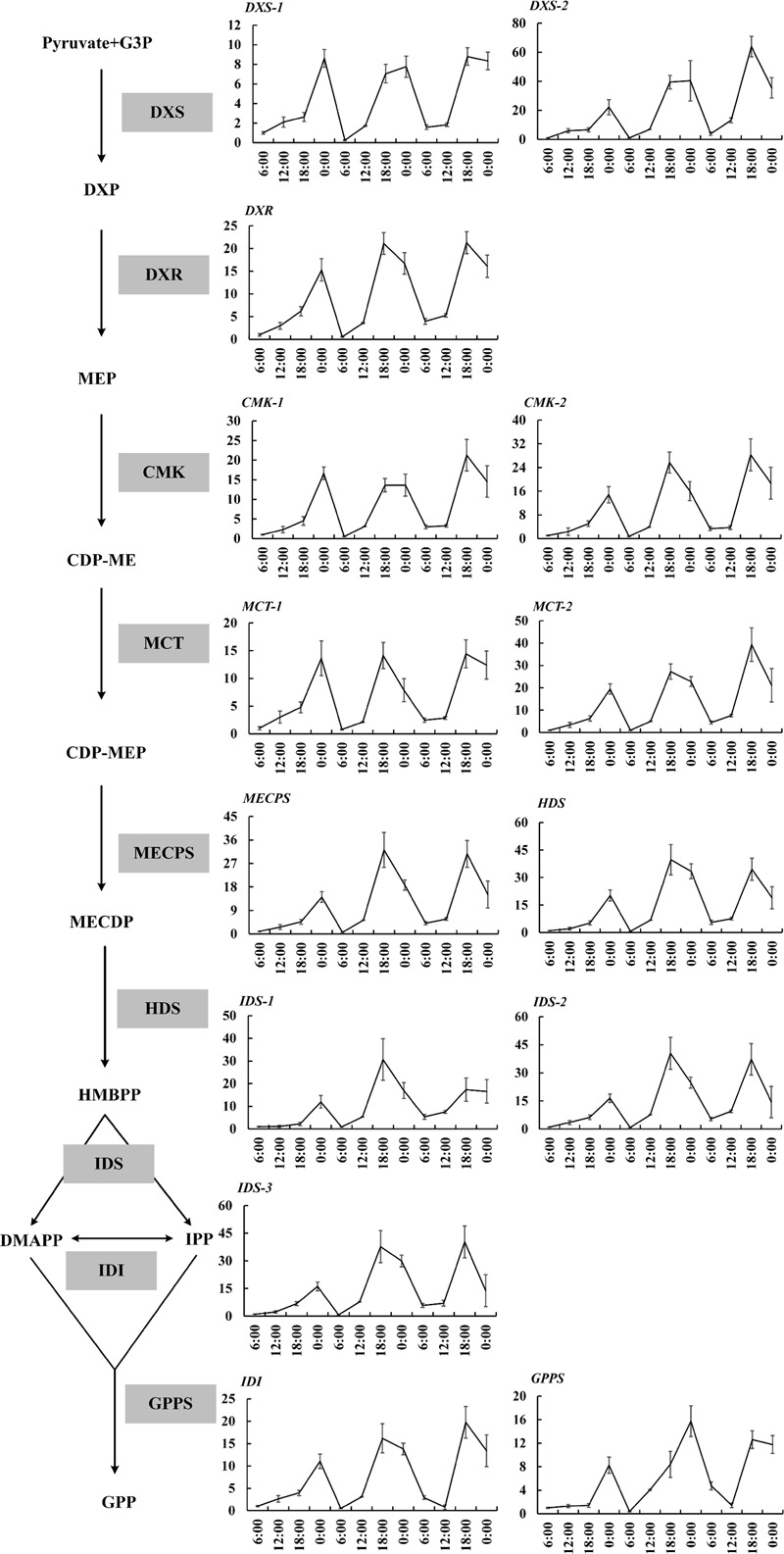
Circadian variation of transcript levels in MEP pathway in *O. fragrans* flowers. mRNA accumulation of genes was measured using qRT-PCR. The relative abundance was determined using a 2^-ΔΔC_T_^ method with β-actin as the reference gene. The data of gene expression at the first 6:00 h was set as 1. Results represent the mean ± SE of three technical repetitions and three biological replicates.

**FIGURE 3:**
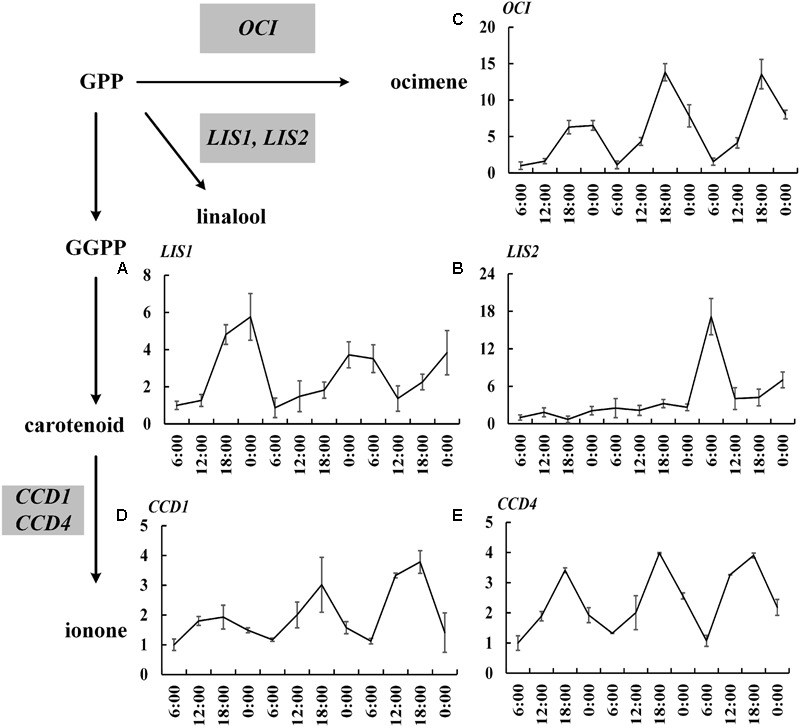
Circadian variation of *OfTPSs, OfCCD1*, and *OfCCD4* transcript levels in *O. fragrans* flowers. **(A)** *OfLIS1*, **(B)** *OfLIS*2, **(C)** *OfOCI*, **(D)** *OfCCD1*, and **(E)** *OfCCD4*. mRNA accumulation of TPS genes was measured using qRT-PCR. The relative abundance was determined using a 2^-ΔΔC_T_^ method with β-actin as the reference gene. The data of gene expression at the first 6:00 h was set as 1. Results represent the mean ± SE of three technical repetitions and three biological replicates.

The expressions of 19 important genes involved in terpenoid formation were detected. The results showed that 14 MEP pathway genes including *DXS1, DXS2, DXR, CMK1, CMK2, MCT1, MCT2, MECPS, HDS, IDS1, IDS2, IDS3, IDI, GPPS* basically confined to uniform circadian oscillation. Their expressions constantly increased during the day (6:00–18:00 h) and sharply decreased during the night (0:00–6:00 h). The maximum expression mainly occurred at 18:00 h and the minimum expression all occurred at 0:00 h (**Figure 2**). The expressions during the night were generally higher than the expression during day and this trend was inconsistent with the volatile compound variation but somehow in line with the free compound variation (**Figures 1D,E, 2**). *OfLIS1* expression also presented a notable increase during evening (18:00–0:00 h) over the whole course which was similar to the MEP pathway genes (**Figure 3**). The promiscuity of *OfLIS2* expression was not synchronized with the normal circadian cycle of emission and/or accumulation of linalool and its derivatives and might indicate it was not a key rate-limiting gene controlling linalool formation (**Figures 1A,E,G, 3**). The notable increase of MEP pathway gene expressions during the day not only obviously accelerate the emission of volatile linalool and its derivatives (6:00–12:00 h) but also significantly promoted the accumulation of non-volatile linalool and its derivatives (6:00–18:00 h) (**Figures 1A,E,G, 2, 3**). We noticed a sharp decline of volatile compounds happened during 12:00–0:00 h repeatedly, suggesting that a large amount of aroma compounds were prone to accumulate as non-volatile conjugates. It was also worth noting that although the MEP pathway genes and *OfLIS1* expressions maintained a constant decrease at 0:00–6:00 h, the volatile compounds displayed a unexpected increase possibly due to the depletion of free and glycosylated compounds. According to these results, we speculated that MEP pathway genes played important roles in formation of linalool and its derivatives and *OfLIS1* was the key rate-limiting factor. The pool of free and glycosylated compounds also exerted influence on the emission of volatile compounds.

Similar variation was also found in the transcript level of *OfOCI, OfCCD1*, and *OfCCD4.* These genes confined to a regular fluctuation with increased expression during the day and decreased expression during the night and always achieved maximum level at 18:00 h (**Figure 3**). It was intriguing that the emission of *trans*-β-ocimene and *cis*-β-ocimene reached maximum value at 12:00 h and declined to minimum value at 18:00 h. The variation of ocimene was even prior to the transcript level of relevant gene suggesting that the substrate supply or other factors might also regulate the rate of compound formation. Consistent with the accumulation of free ionone and its derivatives, the *OfCCD1* and *OfCCD4* expressions increased until reaching peak level at 18:00 h and then declined to low level during the night.

In sum, the circadian expression of relevant genes was highly relative with the circadian oscillation of terpenoid formation. Moreover, the accumulation and depletion of the free and glycosylated compounds pool might act like a buffer modulating the emission of volatile terpenoids.

## Discussion

### Circadian Oscillation of Terpenoid Emission Is Partially Controlled by Rhythmic Gene Expression

The volatile and free terpenoids presented obvious circadian oscillation. Specifically, volatile terpenoids tended to increase quickly at 6:00–12:00 h, coinciding with the significant increase of MEP pathway genes, *TPSs, CCD1*, and *CCD4* expressions. This result demonstrated that the diurnal fluctuations of the terpenoid emission were probably a result of the rhythmic gene expressions. Similar association was also observed in other plants (Dudareva et al., 2005; Loivamäki et al., 2007; Wiberley et al., 2009; Nagegowda et al., 2010). In poplar leaves (Wiberley et al., 2009), the isoprene exhibited significant diurnal oscillation corresponded to changes in mRNA accumulation for several MEP pathway genes and *TPSs* without any changes in TPSs protein. A direct evidence was shown in snapdragon flower investigation, the elimination of the MEP pathway by the inhibitor fosmidomycin led to the elimination of rhythmicity in nerolidol emission. Thus, the diurnal rhythmicity of terpenoid emission in snapdragon flowers was controlled by the flux of MEP pathway (Dudareva et al., 2005). Furthermore, the correlation between specific MEP pathway genes and terpenoid formation was investigated. *DXR* was supposed to be a key point of metabolic flux since it catalyzed the first committed step (Mahmoud and Croteau, 2001; Carretero-Paulet et al., 2006) and had a feedback regulation by IPP and DMAPP supply (Alexandra et al., 2015). *DXS* was also an important rate-limiting player in the pathway (Lois et al., 2000; Estévez et al., 2001).

Most plants emit peak terpenoid volatiles at midday or early in the afternoon regulated by light or internal circadian clock (Dudareva et al., 2003). Three main reasons might account for this phenomenon. First, the two immediate precursors of the MEP pathway, pyruvate and glyceraldehyde 3-phosphate, may be derived directly from the Calvin cycle and closely related to photosynthesis. The study in *A. thaliana* proved that the flux through the MEP pathway could be accelerated by the photoassimilate supply (Alexandra et al., 2015). Even across species, there is a positive correlation between maximum diurnal change in both isoprene emission and photosynthesis (Funk et al., 2003). Second, the sequence of some MEP pathway genes and *TPSs* can respond to light or internal circadian clock. In poplar, the promoter regions of *DXS, CMS, MCS, HDS, HDR*, and *TPSs* all contained a conversed ‘TATTCT’ nucleotides which proved to be light-responsive in *Hordeum vulgare* (Wiberley et al., 2009). The general morning activators CIRCADIAN CLOCK ASSOCIATED 1 (CCA1) and LATE ELONGATED HYPOCOTYL (LHY) can trigger the circadian expression of key MEP pathway genes such as *DXS* and *HDR*, as well as downstream *PSY* (Phillips et al., 2008; Rodríguez-Concepción, 2010). Analysis of these gene sequences also showed the presence of multiple LHY/CCA1-binding elements in the promoter regions in *Arabidopsis* (Alexandra et al., 2015).

### Sequestration of Non-volatile Compounds Is Also an Important Rate-Limiting Player in Regulating Terpenoid Emission

Although the rhythmic gene expressions play important roles in regulating terpenoid emission, it could not explain the whole story. We noticed that despite the significant decrease of MEP pathway genes expressions, the volatile compounds increased obviously from 0:00 to 6:00 h. This prompted us to investigate the non-volatile compounds, the depletion of free and glycosylated terpenoids might transform into volatile terpenoids and well reconciled this conflict. Actually, the emission of volatile compounds into the atmosphere depends on both the rate of biosynthesis and the rate of release (Dudareva et al., 2013). Free and glycosylated compounds commonly coexisted in various flowers and have substantial effect on modulating terpenoid emission (Aharoni et al., 2003; Boachon et al., 2015). Direct evidence came from research on linalool synthase, by overexpression a Clarkia (S)-linalool synthase gene in petunia, unexpected S-linalyl-β-D-glucopyranoside was abundantly detected instead of volatile linalool (Lücker et al., 2001). In addition, we noticed that a cascade of actions took place before glycosylation. Dihydro-3-oxo-β-ionol and 3-oxo-α-ionol were the dominant glycosylated ionone derivatives which could not be detected in volatile and free forms, suggesting that P450 genes had important impact on glycosylation as well as glucosyltransferase genes. A series of promiscuous cytochrome P450 genes have been identified to endow important metabolism on terpenoids and facilitated the production of diverse derivatives for further glycosylation (Luo et al., 2001; Boachon et al., 2015).

### Terpenoids in *O. fragrans* and Their Potential Functions

Terpenoids represent one of the dominant classes of natural aroma compounds and serve different biological functions (Bönisch et al., 2014). *O. fragrans* is a famous plant with pleasant scent by human being olfactory and prolific terpenoids were identified as aroma-active compounds which play crucial roles in formation of the scent bouquet (Cai et al., 2014). Monoterpenes linalool and ocimene produced by MEP pathway and carotenoid-derived ionone provided the primary skeleton for further processing. For example, the double bonds and C-3 hydroxyl group of linalool make it vulnerable to be converted into furanoid and pyranoid linalool oxides (Steenhuisen et al., 2012; Robert, 2016). The furanoid linalool oxides may be further catalyzed to 16 possible stereoisomeric aldehydes and alcohols, collectively known as ‘lilac compounds’ (Dötterl et al., 2006). These compounds with different organoleptic properties usually combined as complex mixtures and constituted the predominant aroma-active components in flowers (Boachon et al., 2015).

The complex bouquet of scent leads us to address an intriguing question that why do *O. fragrans* initiate such complicated reactions to produce diverse derivatives since linalool, β-ionone themselves are considered to be pollinator attraction (Rocha and Garofalo, 2014; Molly and Santiago, 2016). We have to bear two things in mind. First, *O. fragrans* can attract *Vespa nigrithorax, Apis florea* and *Episyrphus balteatus* to visit the flowers to enhance pollination. The observation showed that the visiting usually happened during the day and the peak time was from 9:00 to 12:00 h, no insect visited flowers during the night (Zhang, 2013). The timing is principally parallel with the emission of volatile terpenoids, indicating the bouquet is attractive to pollinators during the day. Second, free and glycosylated terpenoids are accumulative during evening and in late blossoming period (Zeng et al., 2016). Actually, linalool derivatives especially lilac compounds are prone to perform as defense against floral antagonists (Junker et al., 2011; McCallum et al., 2011). Analysis of insect behavior on transgenic *Arabidopsis* flowers showed that the modulation of linalool emission and production of linalool oxides contributed to reduce floral attraction and favor protection against visitors and pests (Boachon et al., 2015). (*E*)-β-ocimene is also one of the most common herbivory-induced plant volatiles and can release as indirect defense strategy in plants (Arimura et al., 2008). Thus, we speculated that by fine-tuning the synthesis and conversion of terpenoids, *O. fragrans* flowers displayed an equilibrium between pollinator attraction and antagonist defense to ultimately serve plant fitness.

In sum, the transcript level of MEP pathway genes along with non-volatile sequestration were closely related with the circadian rhythm of terpenoid emission in O. fragrans flowers. However, the P450 genes for derivative conversion and glycosyltransferase genes for glycosylation have not been revealed in O. fragrans yet. The terpenoid compound analysis and transcriptome sequencing we present here can be a good starting point to elucidate further mechanism of aroma compound emission and conversion in the future.

## Author Contributions

RZ and CW conceived and designed the research. CL, YW, WX, and RZ determined the aroma compound contents. RZ, XZ, YW, and HD analyzed the transcriptome sequences and tested the gene expressions. RZ wrote the manuscript. CW, JL, and JG provided technical guidance and supervised the writing. All authors took part in analyzing the data and approved the manuscript.

## Conflict of Interest Statement

The authors declare that the research was conducted in the absence of any commercial or financial relationships that could be construed as a potential conflict of interest.

## Supplementary Material

FIGURE S1 GO functional classification of the assembled unigenes.

FIGURE S2 KOG functional classification of the assembled unigenes.

FIGURE S3 KEGG functional classification of the assembled unigenes.
