# Supplementary figures and images for: Expression of MEP Pathway Genes and Non-volatile Sequestration Are Associated with Circadian Rhythm of Dominant Terpenoids Emission in Osmanthus fragrans Lour. Flowers
Source: Front Plant Sci. 2017 Oct 30;8:1869. doi: 10.3389/fpls.2017.01869 (PMC5670350; doi:10.3389/fpls.2017.01869)

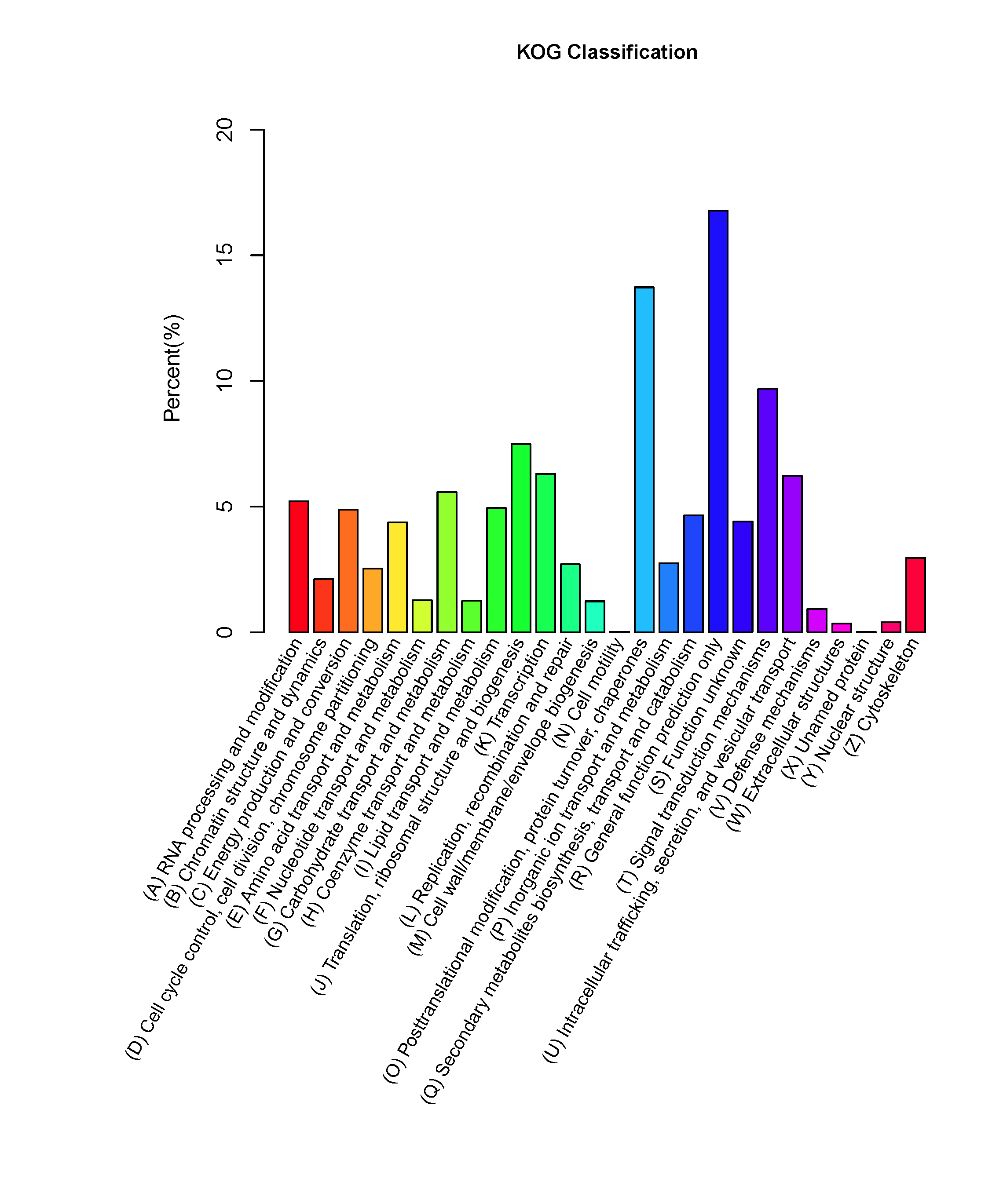

Supplement: FIGURE S2 — KOG functional classification of the assembled unigenes. [file Image_2.tiff]

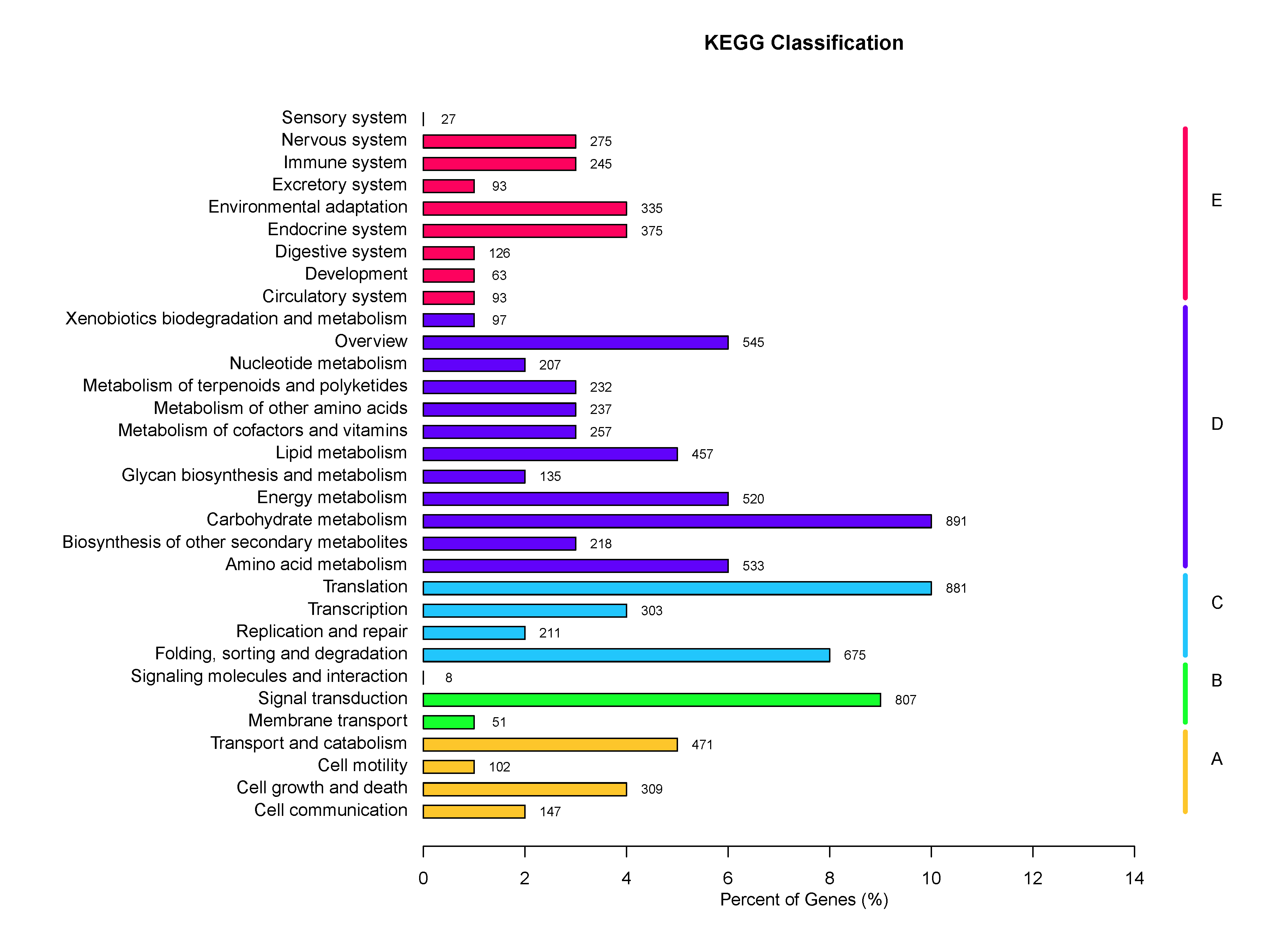

Supplement: FIGURE S3 — KEGG functional classification of the assembled unigenes. [file Image_3.tiff]
