# Supplementary material for: Expression of MEP Pathway Genes and Non-volatile Sequestration Are Associated with Circadian Rhythm of Dominant Terpenoids Emission in Osmanthus fragrans Lour. Flowers
Source: Front Plant Sci. 2017 Oct 30;8:1869. doi: 10.3389/fpls.2017.01869 (PMC5670350; doi:10.3389/fpls.2017.01869)
Supplement: Supplementary file 4 [file Table_1.DOC]

Supplementary Material

**Expression of MEP Pathway Genes and Non-volatile Sequestration Are Associated with Circadian Rhythm of Dominant Terpenoids Emission in *Osmanthus fragrans* Lour. Flowers**

**Riru Zheng*, Cai Liu, Yanli Wang, Jing Luo, Xiangling Zeng, Haiqin Ding, Wei Xiao, Caiyun Wang**

*** Correspondence:** Caiyun Wang: [wangcy@mail.hzau.edu.cn](mailto:wangcy@mail.hzau.edu.cn)

**Table 1** Gene primers used for qRT-PCR analyses

| Primer | Sequence |
| --- | --- |
| *OfDXS*-1-F0  *OfDXS*-1-R0 | 5'-CTATCTCAGCAGGACTAGGAATGGC-3'  5'-GGGTAACGAGACTTGTTTGTTGTCA-3' |
| *OfDXS*-2-F0  *OfDXS*-2-R0 | 5'-ACAGACCAAGCTGCTTTAGGTTTCC-3'  5'-CGGGAATACGTTATGGGATTTTAGC-3' |
| *OfDXR*-F0  *OfDXR*-R0 | 5'-CCATGACAGGGGTTCTTAGCG-3'  5'-CGTAATCCCGAGCCCACAAGT-3' |
| *OfMCT*-1-F0  *OfMCT*-1-R0 | 5'-ACTATCAAAGAGGCAAATGGTGAAT-3'  5'-CACAGGATGTTTAAGGTGCTCTATG-3' |
| *OfMCT*-2-F0  *OfMCT*-2-R0 | 5'-CATCGTCGGTATTCATCAAAGAAGC-3'  5'-ACCAAGTAACTCGCACAAATTAGCC-3' |
| *OfCMK*-1-F0  *OfCMK*-1-R0 | 5'-GGGTTCGATTTTCAACAATCAGAAC-3'  5'-ATCCACTTCATCGGCTAGTTTATTG-3' |
| *OfCMK*-2-F0  *OfCMK*-2-R0 | 5'-TGGTGGTGGTAGTAGTAATGCCG-3'  5'-TGTACAGTAGGCTGCTCCATGAGAG-3' |
| *OfMECPS*-F0  *OfMECPS*-R0 | 5'-CCCGACAATGACCCTAAATGGA-3'  5'-TCTGCACCAAGTAGCTCGCACA-3' |
| *OfHDS*-F0  *OfHDS*-R0 | 5'-GCAGTTGACGAAGCCATTACCC-3'  5'-TCGCCACGCACAGAAACCAC-3' |
| *OfIDS*-1-F0  *OfIDS*-1-R0 | 5'-TTTTGCAGGAAAGTACATCATTGTG-3'  5'-ACCAAGTTTTACAAGATCCGTGTTT-3' |
| *OfIDS*-2-F0  *OfIDS*-2-R0 | 5'-GAGGCAACGTATGTGTGCGATTA-3'  5'-AAACCCCTCGGATACAGCATATTTA-3' |
| *OfIDS*-3-F0  *OfIDS*-3-R0 | 5'-TCGTCGGCTACTCTGGAGTCG-3'  5'-CTTGTGTACTCGCGGTTCATGAG-3' |
| *OfIDI*-F0  *OfIDI*-R0 | 5'-CAGATGCTGGTGAGGGTGGTATTA-3'  5'-TGCTTTGCTCAGAGTCCCTTTCT-3' |
| *OfGPPS*-F0  *OfGPPS*-R0 | 5'-GCTCATGAACACCTGCCAATCA-3'  5'-GGAACCATACCATCTCCCGTAAGA-3' |
| *OfTPS1*-F0  *OfTPS1*-R0 | 5'-GAATATGCTACAGAAAGTGAACACGA-3'  5'-GAAGTCCCACAGTCCATAAAAAGC-3' |
| *OfTPS2*-F0  *OfTPS2*-R0 | 5'-CTATTCTCCGTCTCTGGGATGACTT-3'  5'-TTCACGTAGCACTCTATGTACGAACC-3' |
| *OfTPS3*-F0  *OfTPS3*-R0 | 5'-CTCTACTTCACCCTCCTTAGACAACA-3'  5'-TATTCTCTCCATTATATGCAACACGT-3' |
| *OfTPS4*-F0  *OfTPS4*-R0 | 5'-GGTTTGTCCGTTGCATTTGAGTG-3'  5'-AGATCCTTTTGCTGTTGGGCTTG-3' |
| *OfActin*-F0  *OfActin*-R0 | 5'-ATTAGTCCTCTTCCAGCCTTCTTTG-3'  5'-ATTATTTCCTTGCTCATACGGTCAG-3' |

Table 2 Quality of sequencing.

| Sample | Raw reads | Clean reads | Clean bases | Error(%) | Q20(%) | Q30(%) | GC(%) |
| --- | --- | --- | --- | --- | --- | --- | --- |
| LY-1 | 49995711 | 47761953 | 4.78G | 0.03 | 98.45 | 94.12 | 43.69 |
| LY-2 | 49995711 | 47761953 | 4.78G | 0.03 | 97.38 | 91.92 | 43.74 |

Table 3 Length distribution of transcripts and unigenes of the transcriptome

|  | Min length | Mean length | Median length | Max length | N50 | N90 | Total nucleotides |
| --- | --- | --- | --- | --- | --- | --- | --- |
| Transcripts | 201 | 1005 | 635 | 14089 | 1681 | 405 | 85686167 |
| Unigenes | 201 | 760 | 396 | 14089 | 1385 | 287 | 37835880 |

Table 4 Functional annotation of the transcriptome

| Databases | Number of unigenes | Percentage(%) |
| --- | --- | --- |
| Annotated in NR | 27470 | 55.18 |
| Annotated in NT | 11631 | 23.36 |
| Annotated in KO | 8623 | 17.32 |
| Annotated in SwissProt | 19612 | 39.39 |
| Annotated in PFAM | 18012 | 36.18 |
| Annotated in GO | 20846 | 41.88 |
| Annotated in KOG | 9998 | 20.08 |
| Annotated in all databases | 3510 | 7.05 |
| Annotated in at least one database | 29311 | 58.87 |
| Total unigenes | 49781 | 100 |
